# Establishment and Characterization of Rat Portal Myofibroblast Cell Lines

**DOI:** 10.1371/journal.pone.0121161

**Published:** 2015-03-30

**Authors:** Michel Fausther, Jessica R. Goree, Élise G. Lavoie, Alicia L. Graham, Jean Sévigny, Jonathan A. Dranoff

**Affiliations:** 1 Division of Gastroenterology & Hepatology, University of Arkansas for Medical Sciences, Little Rock, AR, United States of America; 2 Research Service, Central Arkansas VA Healthcare System, Little Rock, AR, United States of America; 3 Département de Microbiologie-Infectiologie et d'Immunologie, Faculté de Médecine, Université Laval, QC, Canada; 4 Centre de Recherche du CHU de Québec, QC, Canada; University of Navarra School of Medicine and Center for Applied Medical Research (CIMA), SPAIN

## Abstract

The major sources of scar-forming myofibroblasts during liver fibrosis are activated hepatic stellate cells (HSC) and portal fibroblasts (PF). In contrast to well-characterized HSC, PF remain understudied and poorly defined. This is largely due to the facts that isolation of rodent PF for functional studies is technically challenging and that PF cell lines had not been established. To address this, we have generated two polyclonal portal myofibroblast cell lines, RGF and RGF-N2. RGF and RGF-N2 were established from primary PF isolated from adult rat livers that underwent culture activation and subsequent SV40-mediated immortalization. Specifically, Ntpdase2/Cd39l1-sorted primary PF were used to generate the RGF-N2 cell line. Both cell lines were functionally characterized by RT-PCR, immunofluorescence, immunoblot and bromodeoxyuridine-based proliferation assay. First, immortalized RGF and RGF-N2 cells are positive for phenotypic myofibroblast markers alpha smooth muscle actin, type I collagen alpha-1, tissue inhibitor of metalloproteinases-1, PF-specific markers elastin, type XV collagen alpha-1 and Ntpdase2/Cd39l1, and mesenchymal cell marker ecto-5’-nucleotidase/Cd73, while negative for HSC-specific markers desmin and lecithin retinol acyltransferase. Second, both RGF and RGF-N2 cell lines are readily transfectable using standard methods. Finally, RGF and RGF-N2 cells attenuate the growth of Mz-ChA-1 cholangiocarcinoma cells in co-culture, as previously demonstrated for primary PF. Immortalized rat portal myofibroblast RGF and RGF-N2 cell lines express typical markers of activated PF-derived myofibroblasts, are suitable for DNA transfection, and can effectively inhibit cholangiocyte proliferation. Both RGF and RGF-N2 cell lines represent novel in vitro cellular models for the functional studies of portal (myo)fibroblasts and their contribution to the progression of liver fibrosis.

## Introduction

Portal fibroblasts (PF) are defined as resident spindle-shaped fibroblasts found in the portal mesenchyme, with a peribiliary distribution[1]. During liver homeostasis, PF are involved in the maintenance of bile duct cell mass and the synthesis of extracellular matrix proteins[2–4]. Following liver injury leading to development of fibrosis, PF undergo myofibroblastic differentiation, phenotypically transitioning from quiescence to an “activated” state[5]. During this critical process, PF acquire contractile properties mainly through expression of lpha-smooth muscle actin (αSMA) and exhibit increased fibrogenic activity through production and release of fibrillar collagens. Expression of αSMA and release of collagen have been seen as indicators of myofibroblastic differentiation/activation. Indeed, recent fate mapping studies clearly indicate that, similar (although to a lesser extent) to hepatic stellate cells (HSC), PF represent cellular precursors of myofibroblasts during liver fibrosis[6,7]. Importantly, the contribution of PF to liver fibrosis is thought to be of particular importance in cholestatic liver injury but less so in hepatocellular injury[8]. However, the functions of PF in liver health and disease remain poorly defined and understudied. In that regard, a contributing factor is certainly the lack of in vitro models for portal (myo)fibroblasts. In contrast, a plethora of in vitro models to study HSC from human and murine species are available: human LX-1[9], LX-2[9], and hTERT[10] cell lines, mouse GRX[11] and JS1[12] cell lines, and rat HSC-T6[13] and CFSC[14] cell lines have been described, yet no immortalized portal (myo)fibroblast have been reported to date. Moreover, primary rodent PF isolation methods remain feasible but challenging due to variability in cell numbers, purity, viability, and growth capacity. To address this issue, we sought to establish PF cell lines via SV40 large T antigen-mediated immortalization of primary isolated rat PF. We describe, in the present report, the generation and characterization of two immortalized rat portal myofibroblast cell lines, RGF and RGF-N2 generated using this approach.

## Methods

### Materials and Reagents

Cell culture reagents and media were obtained from Life Technologies (Life Technologies, Carlsbad, CA). Molecular biology reagents and kits were obtained from Life Technologies and Qiagen (Qiagen, Valencia, CA). All other reagents and chemicals were of the highest quality available.

### Animal Care

All rat experiments were performed in accordance with regulations approved by the University of Arkansas for Medical Sciences Institutional Animal Care and Use Committee. Male Sprague-Dawley rats (12 weeks/400 g) were purchased from Charles River Laboratories (Redfield, AR) and used for two-step collagenase liver perfusion protocol[15,16].

### Isolation of Rat Portal Fibroblasts

Primary PF were isolated from freshly perfused livers of healthy rats, as previously described[15,16]. The liver was perfused through the portal vein with Hank's Balanced Salt Solution (HBSS) minus Ca^2+^/Mg^2+^ buffer (Life Technologies) supplemented with heparin (Fresenius Kabi, Lake Zurich, IL) for blanching. Upon inferior vena cava transection to allow blood and fluid drainage, the liver was further perfused with HBSS buffer minus Ca^2+^/Mg^2+^ alone, then with a collagenase (type 2 blend) solution (Worthington Biochemical, Lakewood, NJ) in HBSS plus Ca^2+^/Mg^2^ buffer. The liver was then removed from the rat and triturated in cold Leibovitz's media (Life Technologies), to tease away parenchymal cells from the biliary tree. The recovered hilar remnants were further digested in a solution of pronase (Roche, Indianapolis, IN) in Dulbecco's modified Eagle's medium/F-12 media (DMEM/F-12, Life Technologies) supplemented with type 2 collagenase and DNAse (Sigma-Aldrich), followed by mesh filtration (40-micron cell strainer, Corning Life Sciences, Tewksbury, MA). The remaining pronase-resistant hilar remnants were recovered and further digested in a solution of hyaluronidase (Sigma-Aldrich, St-Louis, MO) in DMEM/F-12 media supplemented with type 2 collagenase and DNAse, again followed by mesh filtration. The liberated cells from both pronase and hyaluronidase digestion steps were combined and washed with a solution of DNAse in RPMI 1640 media (Life Technologies). Finally, the resulting cell suspensions of non-parenchymal cells (mainly portal fibroblasts) were plated in medium containing Dulbecco's modified Eagle's medium/F-12 supplemented with 10% fetal bovine serum, 2% penicillin-streptomycin, 0.3% gentamycin, and 0.1% fungizone (Life Technologies).

### Immortalization of Rat Portal Fibroblasts

Primary isolated rat PF were grown on plastic for 2 days before media change, and then passaged every 4 days twice, at which time cell purity approaches 100% and myofibroblastic differentiation is observed (S1 Fig., *PRIMARY PF*)[15]. To immortalize primary isolated portal myofibroblasts, cells were transfected with 5 micrograms of a SV40 large T Antigen mammalian expression vector (Addgene plasmid #21826 generated by Dr. David Ron, and obtained from Addgene, Cambridge, MA) using Fugene6 transfection reagent (Promega, Madison, WI). After transfection, cells were grown to confluence and then serially passaged for selection. Cell pools were expanded into RGF and RGF-N2 cell lines and passaged at least 45 times, before analysis (S1 Fig., *RGF* and *RGF-N2*). In the case of RGF-N2 cell line, primary isolated cells were sorted before plating and immortalization, using a rabbit polyclonal antibody raised against PF marker rat Ntpdase2 (rN2-6_L_, Ectonucleotidases-Ab, Quebec, QC, Canada)[17] pre-labeled with Alexa Fluor 488 dye from a Zenon Rabbit IgG Labeling Kit (Life Technologies) according to the manufacturer’s instructions alone, or in combination with a mouse monoclonal antibody raised against rat Thy1.1+[18] coupled to PerCP-Cy5.5 dye (clone HIS51, Life Technologies) (S2 Fig.). In all sorting experiments, propidium iodide (Life Technologies) was used for live cell labeling.

### Gene Expression Analysis

**Fig 1:**
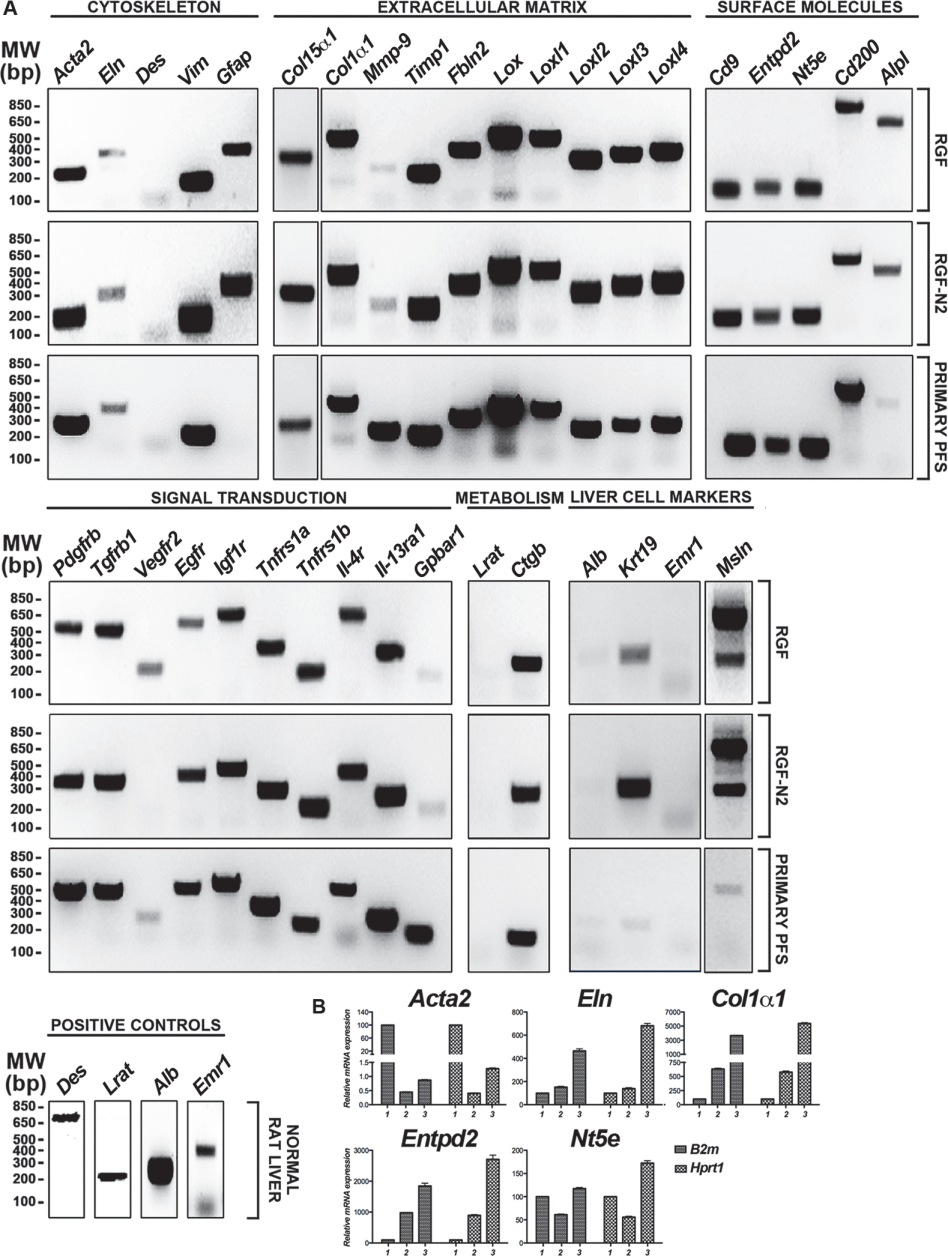
Phenotypic characterization of immortalized rat portal fibroblastic RGF and RGF-N2 cell lines by RT-PCR. (A) Cultured primary isolated aPF (8 days) and immortalized rat PF RGF and RGF-N2 cDNA samples were used as templates in PCR reactions with primers specific to transcripts of several fibrogenic genes in liver myofibroblasts (see Table 1 for gene list and accession numbers). RGF and RGF-N2 cell lines express classical αSMA (*Acta2*), type I collagen α1 (*Col1*α*1*) liver myofibroblast gene products, along with PF-derived elastin (*Eln*), type XV collagen α1 (*Col15*α*1*), and Ntpdase2 (*Entpd2*) myofibroblast markers, also found in primary culture-activated portal myofibroblasts. Both cell lines are devoid of HSC-derived myofibroblastic desmin (*Des*) and lecithin-retinol acyl transferase (*Lrat*) markers. Rat normal liver or brain cDNA was used as positive control, when no PCR amplicon was detected (not shown). Nuclease-free water was used as negative control (not shown). *MW*, *molecular weight; bp*, *base pairs*. (B) Cultured primary isolated aPF (8 days, 2 primary cell isolations) and immortalized rat PF RGF (passages #61 and #64) and RGF-N2 cDNA (passages #54 and #55) samples were used as templates in quantitative PCR reactions with probes specific to transcripts of fibrogenic *Acta2*, *Eln*, *Col1*α*1*, *Entpd2*, and *Nt5e* genes in liver myofibroblasts. Housekeeping *B2m* and *Hprt1* genes were separately used as references.

Total RNA extractions were performed from rat liver tissue, primary isolated PFs, and SV40-immortalized RGF and RGF-N2 PFs, using a RNeasy Plus Kit (Qiagen). Quantification of total RNA concentration was performed using the Qubit RNA Assay Kit with a Qubit 2.0 Fluorometer (Life Technologies). Treatment of isolated total RNA samples with RNAse-free Ambion DNAse1 enzyme (Life Technologies) was performed to remove any genomic DNA contamination. Complementary DNA (cDNA) synthesis reaction was performed using the iScript Reverse Transcription Supermix (Bio-Rad Laboratories, Hercules, CA). Primary and immortalized cell cDNA reaction products were further used as templates for polymerase chain reaction (PCR) amplification with the TopTaq Master Mix Kit (Qiagen). Target-specific PCR primers sets (Integrated DNA Technologies—IDT, Coralville, IA) were designed using NCBI’s Primer-BLAST program[19] and are listed in Table 1. The following PCR protocol was used: initial denaturation step at 94°C for 3 minutes, followed 35 repetitions of a 3-step cycling program consisting of 30 seconds at 94°C (denaturation), 30 seconds at 60°C (primer annealing), and 30 seconds at 72°C (elongation), and final extension step of 10 minutes at 72°Celsius. Amplification products were visualized on 1% (w/v) agarose (Life Technologies) gels containing 0.5 microgram per milliliter of ethidium bromide nucleic acid stain (Bio-Rad Laboratories) (Fig. 1). Primary and immortalized cell cDNA reaction products (dilution 1:5 in nuclease-free water) were also used as templates for quantitative PCR amplification with the SsoAdvanced Universal Real-Time PCR Supermix (Bio-Rad Laboratories) and validated specific probes for *Acta2* (αSMA, Taqman#Rn01759928_g1), *Eln* (Elastin, IDT#Rn.PT.58.8283578), *Col1α1* (type I collagen alpha-1, IDT#Rn.PT.58.11414207), *Entpd2* (ectonucleoside triphosphate diphosphohydrolase 2, IDT#Rn.PT.58.37204991.g), *Nt5e* (ecto-5’-nucleotidase, IDT#Rn.PT.58.14072388), and housekeeping *B2m* (beta-2-microglobulin, DT#Rn.PT.58.11709934) and *Hprt1* (hypoxanthine phosphoribosyltransferase 1, IDT#Rn.PT.39a.22214832) genes in rat species. The following PCR protocol was used: initial denaturation step at 95°C for 3 minutes, followed 40 repetitions of a 2-step cycling program consisting of 10 seconds at 95°C, 30 seconds at 55 or 57°C.

**Table 1:** Sequences of primer sets used for gene expression analysis by PCR.

GENE NAME	ACCESSION NUMBER	PRIMER SEQUENCES	PRODUCT SIZE (BP)
**CYTOSKELETON**			
**Alpha-smooth muscle actin (*αSMA*)**	**NM_031004.2**	**5’-GCCATCAGGAACCTCGAGAA-3’ 5’-AGTTGGTGATGATGCCGTGT-3’**	**273**
**Elastin (*Eln*)**	**NM_012722.1**	**5’-CAGGAGTCAAGGCCAAGGTT-3’ 5’-CTGGTCCACCAGGCACTAAG-3’**	**447**
**Desmin (*Des*)**	**NM_022531.1**	**5’-CCAGGCCTACTCGTCCA-3’ 5’-GGTCAATTCGAGCCAGAGTG-3’**	**665**
**Vimentin (*Vim*)**	**NM_031140.1**	**5’-TCCTTCGAAGCCATGTCCAC-3’ 5’-GGACGAGGAATAGAGGCTGC-3’**	**180**
**Glial fibrillary acidic protein (*Gfap*)**	**NM_017009.2**	**5’-CTCCCTGTCTCGAATGACGC-3’ 5’-GCGACTCAACCTTCCTCTCC-3’**	**467**
**EXTRACELLULAR MATRIX**			
**Type XV collagen alpha-1 (*Col15α1*)**	**NM_001100535.1**	**5’-CGTGTCCGAGATGGTTGGAA-3’ 5’- CCGCACAACTGTGGAGAGAT-3’**	**297**
**Type I collagen alpha-1 (*Col1α1*)**	**NM_053304.1**	**5’-CAATCTGGTTCCCTCCCACC-3’ 5’-CAGCACAGGCCCTCAAAAAC-3’**	**627**
**Matrix metallopeptidase-9 (*Mmp-9*)**	**NM_031055.1**	**5’-GGCCCCAGGAGTCTGGATAA-3’ 5’-GGTTGTGGAAACTCACACGC-3’**	**271**
**Timp metallopeptidase inhibitor 1 (*Timp1*)**	**NM_053819.1**	**5’-AGAGCAGATACCACGATGGC-3’ 5’-AGCGTCGAATCCTTTGAGCA-3’**	**237**
**Fibulin-2 (*Fbln2*)**	**XM_006224990.1**	**5’-GATACCTGTGGGGTCTCCCT-3’ 5’-GACTCTCGTGCAGTGTCCAA-3’**	**455**
**Lysyl oxidase (*Lox*)**	**NM_017061.2**	**5’-GGCACCGACCTGGATATGGCACC-3’ 5’-CGGTGAAATGGTGCAGCCTGAGG-3’**	**656**
**Lysyl oxidase-like 1 (*Loxl1*)**	**NM_001012125.1**	**5’-CGTCGTTACTCGCATAGCCT-3’ 5’-CCATGCTGTGGTAATGTTGGTG-3’**	**610**
**Lysyl oxidase-like 2 (*Loxl2*)**	**NM_001106047.2**	**5’-AGCCTATAAGCCGGAGCAAC-3’ 5’-GGCGCACCTTTTTCTGGAAG-3’**	**359**
**Lysyl oxidase-like 3 (*Loxl3*)**	**NM_001107866.2**	**5’-TTGGACCCACAGTGCCAAAT-3’ 5’-TGGGCCAGCATCCTGTAGAA-3’**	**426**
**Lysyl oxidase-like 4 (*Loxl4*)**	**NM_001107592.1**	**5’-CTGCGCTTCTCCTCACAGAT-3’ 5’-GGATCTCCTGTGTGGCAGTTG-3’**	**464**
**SURFACE MOLECULES**			
**Cd9 molecule (*Cd9*)**	**NM_053018.1**	**5’-TTGGACTATGGCTGCGGTTC-3’ 5’-GCAGCCCAGGAAACCAACTA-3’**	**137**
**Ectonucleoside triphosphate diphosphohydrolase 2 (*Entpd2*)**	**NM_172030.1**	**5’-CGGACAAGGAGAATGACACA-3’ 5’-TCTCTGGGTACATCCCGAAG-3’**	**151**
**5' nucleotidase, ecto (*Nt5e*)**	**NM_021576.2**	**5’-AGAGCAAACCAGCGATGACT-3’ 5’-GATGGTGCCCTGGTACTGAT-3’**	**151**
**Cd200 molecule (*Cd200*)**	**NM_031518.2**	**5’-GATGGGCAGTCCGGTATTCA-3’ 5’-CCCTCACAGGCTTCCTTCTG-3’**	**980**
**Alkaline phosphatase, liver/bone/kidney (*Alpl*)**	**NM_013059.1**	**5’-CTCTCCAAGACGTACAACACCAA-3’ 5’-ATGGTGCCCGTGGTCAAT-3’**	**735**
**SIGNAL TRANSDUCTION**			
**Platelet-derived growth factor receptor, beta polypeptide (*Pdgfrβ*)**	**NM_031525.1**	**5’- ATCCCAGATACACCCCACGA3’ 5’-TGTGAGCAGTATTCCCCAGC-3’**	**521**
**Transforming growth factor beta, receptor 1 (*Tgfβr1*)**	**NM_012775.2**	**5’-AACATGCACACCCCCAAGAT-3’ 5’-CAGGGCCTCAAGGCACTTTT-3’**	**521**
**Vascular endothelium growth factor receptor 2 (*Vegfr2*)**	**NM_013062.1**	**5’-TAGCGGGATGAAATCTTTGG-3’ 5’-TTGGTGAGGATGACCGTGTA-3’**	**207**
**Epidermal growth factor receptor (*Egfr*)**	**NM_031507.1**	**5’-CACCAAGACAGGCGACGG-3’ 5’-AGCACCGATCAGAATTTCCTGT-3’**	**587**
**Insulin-like growth factor-1 receptor (*Igf1r*)**	**NM_052807.2**	**5’-ATCCGGCGAGGCAATAACAT-3’ 5’-ACGGATGTGGTCGTTTTCCA-3’**	**696**
**Tumor necrosis factor receptor superfamily, member 1a (*Tnfrs1a*)**	**NM_013091.1**	**5’-TGGAGGACCGTACCCTGATT-3’ 5’-TTCCTTTGTGGCACTTGGTG-3’**	**375**
**Tumor necrosis factor receptor superfamily, member 1b (*Tnfrs1b*)**	**NM_130426.4**	**5’-GCCAAACTCCACACATCCCT-3’ 5’-GGCACCATGGTTTCTCGTTG-3’**	**199**
**Interleukin 4 receptor (*Il4r*)**	**NM_133380.2**	**5’-AACACACAGGTGCTGGAGAGG-3’ 5’-GGTGTTGACTGGGAAGCTCA-3’**	**677**
**Interleukin 13 receptor, α1 (Il13ra1)**	**NM_145789.2**	**5’-TGAGTCTGCTGTGACCGAAC-3’ 5’-GGAGGACCGGGTTTCACATT-3’**	**315**
**G protein-coupled bile acid receptor 1 (*Gpbar1*)**	**NM_177936.1**	**5’-TCAGTCTTGGCCTATGAGCG-3’ 5’-CTTGTAGCCACCTTTGGGCA-3’**	**163**
**METABOLISM**			
**Lecithin-retinol acyltransferase (*Lrat*)**	**NM_022280.2**	**5’-TGGTCTCCAACAAGCGTCTC-3’ 5’-AGTAGGCTGTAGGGGGTCAG-3’**	**199**
**Cytoglobin (*Ctgb*)**	**NM_130744.2**	**5’-CCCTCAAGCACAAGGTGGAA-3’ 5’-AAGTCAGCCTTCTGCCCAAA-3’**	**286**
**LIVER CELL MARKERS (NON-FIBROBLASTIC ORIGIN)**			
**Albumin (*Alb*)**	**NM_134326.2**	**5’-GTGAGCGAGAAGGTCACCAA-3’ 5’-CCTTGCAACACTTGTCCACG-3’**	**277**
**Keratin 19 (*Krt19*)**	**NM_199498.1**	**5’-AGGACGCGGTGGAAGTTTTA-3’ 5’-TGGAGTTCTCAATGGTGGCG-3’**	**272**
**EGF-like module containing mucin-like hormone receptor-like 1**	**NM_001007557.1**	**5’-CCTTCCTGTTGTTTCGTGCAG-3’ 5’-ATGATCATGCAGACTGGCCC-3’**	**418**
**Mesothelin (*Msln*)**	**NM_031658.1**	**5’-GTGGTGTGAGTTGAGGGGTG-3’ 5’-GGGATGCTGTGGACAATGGA-3’**	**857**

### Immunoblot

**Fig 2:**
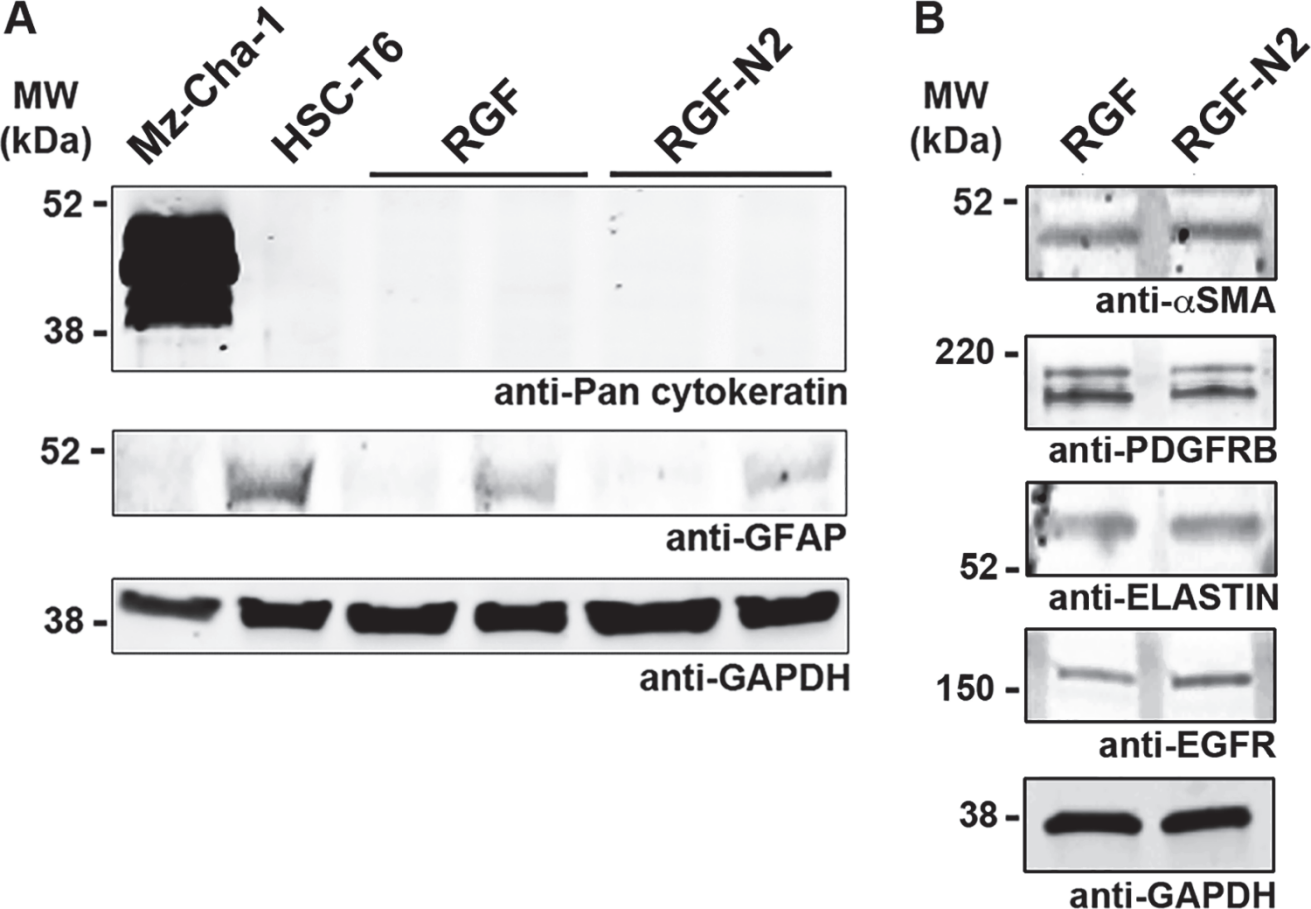
Phenotypic characterization of immortalized rat portal fibroblastic RGF and RGF-N2 cell lines by immunoblot. (A) Immortalized rat PF RGF and RGF-N2 protein samples were analyzed for expression of Gfap and cytokeratins. Housekeeping Gapdh protein was used as loading control, Mz-Cha-1 cell lysate as positive control for cytokeratin expression, and HSC-T6 cell lysate as positive control for Gfap expression. RGF (passage #59, 1^st^ lane and passage #34, 2^nd^ lane) and RGF-N2 (passage #52, 1^st^ lane and passage #25, 2^nd^ lane) cells do express neither cytokeratin proteins nor Gfap protein (after 45 passages for the latter). (B) Immortalized rat PF RGF and RGF-N2 protein samples were analyzed for expression of αSMA, Elastin, Pdgfrβ and Egfr. RGF and RGF-N2 express all proteins tested. Housekeeping Gapdh protein was used as loading control. *kDa*, *kiloDaltons*.

Immortalized rat HSC-T6 liver stellate cells[13], and human Mz-Cha-1 cholangiocarcinoma cells[20] cells were grown as described previously. Confluent RGF, RGF-N2, HSC-T6 and Mz-Cha-1 cells were scraped and lysed with RIPA buffer (Thermo Scientific, Rockford, IL) supplemented with Halt Protease and Phosphatase Inhibitor cocktails (Thermo Scientific) for 5 min. Cell lysates were obtained after centrifugation at 14,000x *g* for 15 min, and protein concentration was determined with the BCA Protein Assay kit (Thermo Scientific) using bovine serum albumin (BSA) as standard. Proteins (40 g per well) were resolved by SDS-PAGE under reducing conditions, and transferred onto a polyvinylidene difluoride membrane (Immobilon/Millipore, Bedford, MA). Membranes were blocked with 5% Carnation skimmed milk in 1X Phosphate-Buffered Saline (PBS) or 1X Tris-Buffered Saline (TBS), incubated with the following primary antibodies (diluted in 5% BSA in 1X PBS or 1X TBS): mouse monoclonal anti-pan cytokeratin (clone C-11, Sigma-Aldrich) and anti-glial fibrillary acidic protein (clone GA5, Cell Signaling, Danvers, MA) antibodies or rabbit polyclonal: anti-αSMA (ab#5694, Abcam, Cambridge, MA), anti-elastin antibody (CL55041AP, Cedarlane Immunologicals), anti-platelet derived growth factor receptor, beta polypeptide (clone#958, Abcam), anti-epidermal growth factor receptor (#PA1-1110, Thermo Scientific), and anti-glyceraldehyde-3-phosphate dehydrogenase (#G9545, Sigma-Aldrich) antibodies, followed by appropriate Molecular Probes goat Alexa Fluor-conjugated anti-mouse IgG and Fluor-conjugated anti-rabbit IgG antibodies (diluted in 0.5% Milk in 1X PBS or 1X TBS), and bands were visualized by use of a Typhoon imaging system (GE Healthcare Bio-Sciences, Pittsburgh, PA) (Fig. 2).

### Immunofluorescence Microscopy

**Fig 3:**
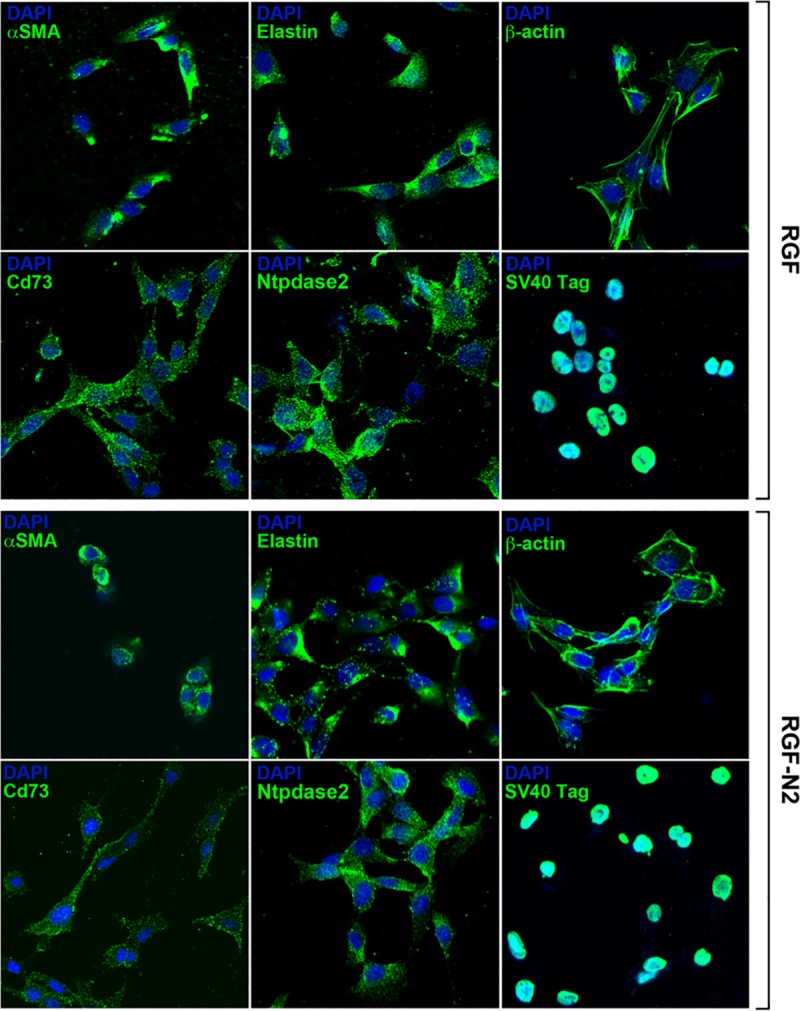
Phenotypic characterization of immortalized rat portal fibroblastic RGF and RGF-N2 cell lines by immunofluorescence. Immortalized RGF cells were fixed, stained with antibodies to proteins specifically expressed in PF-derived myofibroblasts, and counterstained with DAPI nuclear labeling dye. RGF cells express myofibroblast-specific αSMA and Cd73 proteins, PF-specific elastin and Ntpdase2 proteins, β-actin protein and SV40 antigen. Immortalized RGF-N2 cells were fixed, stained with the same antibodies, and counterstained with DAPI dye, as described above for RGF cells. Like RGF cells, RGF-N2 cells express αSMA, Cd73, Elastin, Ntpdase2, β-actin, and SV40 antigen. *400X magnification*.

Immortalized rat RGF and RGF-N2 PF were fixed with neutral 4% paraformaldehyde (PFA) solution (diluted in 1X PBS, pH = 7.2) for 15 min at room temperature and washed in 1X PBS. Fixed cells were then incubated with the following mouse monoclonal antibodies: anti-β-actin (clone AC15, Sigma-Aldrich), anti-rat Cd73 (clone 5F/B9, BD Pharmingen, San Diego, CA), anti-αSMA (clone 1B4, Sigma-Aldrich), anti-simian virus SV40 T antigen (clone Pab101, Santa Cruz Biotechnology, Dallas, TX), and rabbit polyclonal antibodies: anti-rat Ntpdase2 (rN2-6_L_, Ectonucleotidases-Ab) and anti-rat elastin (CL55041AP, Cedarlane Immunologicals, Westbury, NY), overnight at 4°C. Slides were washed 3 times with 1X PBS, then incubated with appropriate Molecular Probes goat Alexa Fluor-conjugated anti-mouse IgG and anti-rabbit IgG antibodies (Life Technologies) for 1 h at room temperature. Slides were washed 3 times with 1X PBS, 1 time with deionized water, and subsequently mounted in ProLong Gold Anti-fade reagent with 4,6-diamidino-2-phenylindole (DAPI) nuclear stain medium (Life Technologies). Slides incubated with secondary antibody alone were used as a control for specificity of fluorescence detection. Confocal fluorescence microscopy images were acquired using Zeiss LSM 510 Meta imaging system (Zeiss Laboratories, White Plains, NY) (Fig. 3).

### Transfection Assay

**Fig 4:**
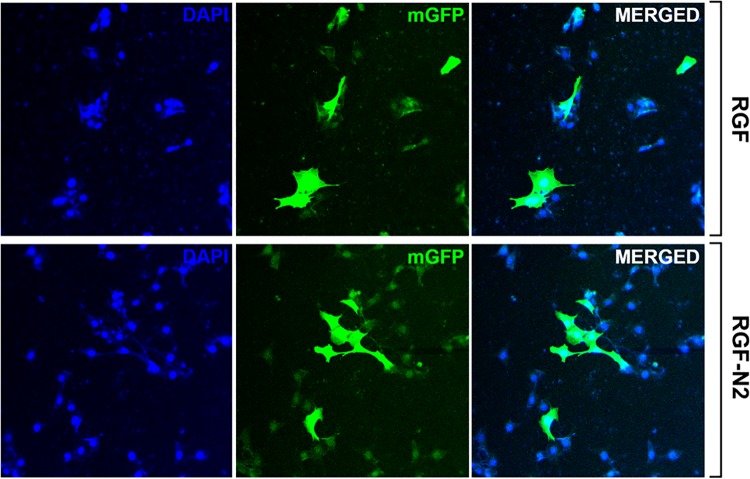
Plasmid DNA transfer in immortalized rat portal fibroblastic RGF and RGF-N2 cell lines by immunofluorescence. RGF and RGF-N2 cells were transfected with an expression vector encoding monomeric GFP protein cDNA using Fugene6 transfection reagent. Transfected cells were then fixed and counterstained with DAPI dye, before confocal microscopy imaging. Both cell lines exhibit green fluorescence signal, indicative of recombinant GFP protein expression. *200X magnification*.

Immortalized RGF and RGF-N2 cells (2,5 x 10^4^) were transfected immediately after trypsinization with a monomeric GFP (mGFP) mammalian expression vector (Addgene plasmid #18696, generated by Dr. Karel Svoboda) using Fugene6 transfection reagent (Promega) at 1:3 (microgram(s) of DNA/microliter(s) of reagent) ratio or Lipofectamine2000 at 1:6 ratio, and grown for 48 h. Transfected cells were then fixed in neutral 4% PFA solution, washed in 1X PBS, and mounted in ProLong Gold Anti-fade reagent with DAPI, prior to confocal fluorescence microscopy imaging (Fig. 4).

### Co-Culture Assay

**Fig 5:**
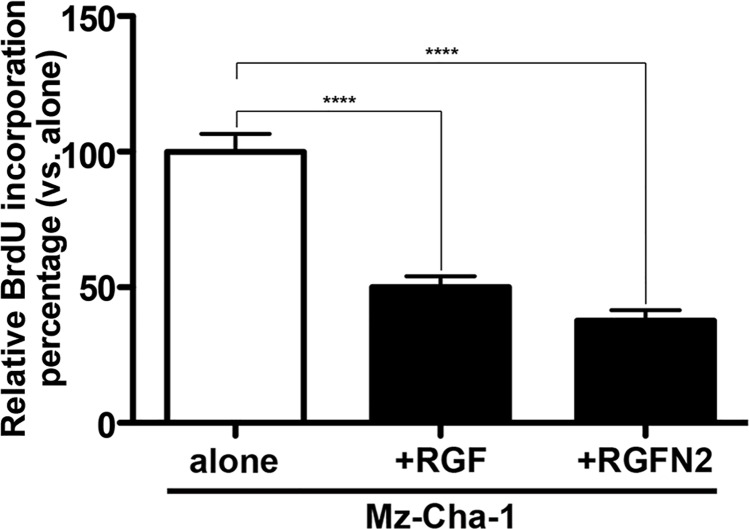
Co-culture of Mz-Cha-1 cholangiocytes with immortalized rat portal fibroblastic RGF and RGF-N2 cell lines by bromodeoxyuridine incorporation assay. Sub-confluent immortalized human Mz-Cha-1 cholangiocytes were labeled with bromodeoxyuridine reagent for 24 hours (day 1), and co-cultured with RGF and RGF-N2 cells for additional 24 hours (day 2), before assessment of bromodeoxyuridine incorporation. Both RGF (****+*RGF*: *M = 50*.*11*, *SE = 3*.*899*, *vs*. *alone*: *M = 100*, *SE = 22*.*76*, *p˂*. *0001*, n = 4) and RGF-N2 (****+RGF-N2: *M = 37*.*64*, *SE = 13*.*40 vs*. *alone*: *M = 100*, *SE = 22*.*76*, *p˂*. *0001*, n = 4) cell lines are able to inhibit proliferation of cholangiocytes.

Immortalized Mz-ChA-1 cells were grown in DMEM-high glucose supplemented with 10% fetal bovine serum, 2% penicillin-streptomycin and used for co-culture assays with RGF or RGF-N2 cells (n = 4 for each PF cell line), as previously described[21]. All cell lines were plated and grown to confluence in individual cell culture dishes, before use. On day 1, Mz-ChA-1 cells (10 x 10^3^ cells count per well, in triplicate) were plated in individual wells of 6-well plates and overlaid with culture media containing bromodeoxyuridine-labeling reagent from a commercial cell proliferation kit (Roche Diagnostics, Indianapolis, IN) for a 24-hour period, to allow incorporation. On day 2, RGF and RGF-N2 cells were added (20 x 10^3^ cells count per well) at an estimated 2:1 ratio to RGF and RGF-N2 cells, as previously described. Both Mz-ChA-1 and RGF or RGF-N2 cells were maintained in co-culture for an additional 24-hour period, in DMEM-high glucose supplemented with 10% fetal calf serum, 2% penicillin-streptomycin. On day 3, proliferation rate of Mz-ChA-1 cells was assessed by ELISA, according to the manufacturer’s instructions (Roche Biosciences). Primary isolated rat PFs were used as experimental control cells (not shown). Labeled Mz-ChA-1 cells grown alone were included as baseline controls (Fig. 5).

### Statistical methods

Statistical analysis was performed using one-way ANOVA followed by Bonferroni's post-hoc multiple comparisons test. *P-value* was considered significant, when P < 0.05.

## Results

To generate the immortalized rat PF cell lines, we first isolated primary rat PF from livers of healthy rats. Primary isolated cells were either immediately plated in the case of RGF cell line, or used for cell staining/sorting before plating in the case of RGF-N2 cell line. Based on published literature, ectonucleoside triphosphate diphosphohydrolase 2/Ntpdase2 (*Entpd2*/*Cd39l1*) and Thy1.1+ (*Cd90*.*1*/*Thy1a*) markers were used for cell sorting and enrichment of PF from primary isolated cell preparation pools[17,18]. In one set of experiments, sorting was performed using an Alexa 488 Zenon-coupled rabbit polyclonal antibody directed against rat Ntpdase2, which selectively stained approximately one third of primary isolated cell preparation (S2 Fig., *NTPDase2*) and, an Alexa 647-conjugated mouse monoclonal anti-rat Thy1.1^+^, which selectively stained approximately less than 4% of primary isolated cell preparation (S2 Fig., *THY1*.*1+*). Both non-sorted and Ntpdase2-sorted primary isolated PF cell preparations were cultured on plastic for 10 days, before transfection with SV40 large T antigen expression vector was performed. Polyclonal RGF and RGF-N2 cell lines were obtained by serial dilutions (1:10, 10 times) and, then, constant passaging steps. Data presented here are from RGF and RGF-N2 cells after 45 passages.

First, RGF and RGF-N2 cells were analyzed and compared to primary isolated activated PF (aPF) for mRNA expression of typical myofibroblastic markers (Fig. 1A and 1B). Like primary isolated aPF cells, both RGF and RGF-N2 cells express classical hepatic myofibroblast markers, extracellular matrix constituent type I collagen alpha-1 (*Col1α 1*)[4], and microfilament–related *α*SMA isoform (*Acta2*)[22,23], tissue inhibitor of metalloproteinase (*Timp1*)[24] mRNAs (Fig. 1A). Primary isolated aPF cells together with RGF and RGF-N2 cells produce mRNAs for PF-specific elastin (*Eln*)[5,25], fibulin-2 (*Fbln2*)[26], type XV collagen alpha-1 (*Col15α 1*)[27], lysyl oxidase (*Lox*), and lysyl oxidase-like 1–4 (*Loxl1-4*)[28] markers. Mmp-9 mRNA expression was robust in primary isolated aPF cells but weak in both RGF and RGF-N2 cell lines. In addition, expression of specific markers for liver myofibroblasts such as, Cytoglobin (*Cygb*)[29], along with intermediate filaments Vimentin (*Vim*)[30], Glial fibrillary acidic protein (Gfap)[31] mRNAs was observed, the latter being absent in primary isolated aPF cells. Importantly, primary isolated aPF, RGF, and RGF-N2 cells lack expression (or express negligible levels) of established hepatic stellate cell (HSC)-specific Desmin (*Des*) and Lecithin-retinol acyltransferase (*Lrat*)[7] markers. Expression of purinergic cell-surface Ntpdase2, Ecto-5’-nucleotidase/Cd73 *(Nt5e)* and Tissue non-specific alkaline phosphatase (*Alpl*) enzymes mRNAs was also detected. Both RGF and RGF-N2 also express several key receptors for signal transduction in myofibroblasts[8,32] including Platelet-derived growth factor receptor, beta polypeptide (*Pdgfrβ*/*Cd140b*), Transforming growth factor, beta receptor 1 (*Tgfβr1*/*Alk5*), Epidermal growth factor receptor (*Egfr*), Insulin growth factor 1 receptor (*Igf1r*/*Cd221*), and Tumor necrosis factor receptor superfamily 1a (*Tnfrs1a*/*Cd120a*) and 1b (*Tnfrs1b*/*Cd120b*) members. Moreover, Interleukin-4 receptor alpha (*Il-4ra*/*Cd124*), Interleukin-13 receptor, alpha 1 (*Il-13ra*/*Cd213A1*), Cd200 (*Mox2*), and Cd9 gene products were also detected. Interestingly, expression of Vascular endothelium growth factor receptor-2 (*Vegfr2*/*Cd309*) appears to be weakly detected or absent in RGF-N2 cells, when compared to RGF cells and primary isolated aPF cells. Expression of G protein-coupled bile acid receptor 1 (*Gpbar1/Tgr5*) mRNA was observed in primary isolated aPF cells but not in RGF and RGF-N2 cells. Separately, expression of markers for other parenchymal and non-parenchymal liver cell types was studied, and showed that RGF and RGF-N2 cells express neither Albumin (*Alb*, hepatocytes) nor F4/80 (*Emr1*, macrophages) markers mRNAs, although Keratin 19 (*Krt19*, cholangiocytes) mRNA expression was detected (Fig. 1A). Expression of Mesothelin mRNA was also observed in both RGF and RGF-N2 cell lines with amplification of an additional splice variant that was unexpected (Fig. 1A). In addition, we studied and compared key fibrogenic portal myofibroblast genes such as, *Acta2*, *Eln*, *Col1α1*, *Entpd2*, and *Nt5e* by quantitative RT-PCR (Fig. 1B). We found that SV40-mediated immortalization did not significantly alter the expression of the analyzed genes, except for *Acta2* gene that was decreased in both cell lines (approximately 200 times for RGF, and 100 times for RGF-N2) relatively to primary isolated aPF, nonetheless with still detectable expression. Second, the cellular origin or identity of RGF and RGF-N2 cell lines was further established, following analysis of total protein extract samples by immunoblot for expression of several proteins including simple epithelium keratins, Gfap, *α*SMA, Elastin, Pdgfrβ and Egfr (Fig. 2). Immunoblot assays show that RGF and RGF-N2 cells express no cytokeratin proteins, suggesting that these cell lines unambiguously derive from hepatic cells distinct from cholangiocytes (Fig. 2A). Additionally, Gfap protein expression was gradually lost with continuous passaging steps (Fig. 2A). Protein expression of myofibroblast-specific *α*SMA and Pdgfrβ markers was observed, as well as the portal (myo)fibroblast-specific marker Elastin (Fig. 2B). Furthermore, immunofluorescence microscopy experiments show that RGF and RGF-N2 cells express *α*SMA and Elastin within the cytoplasm, and Ntpdase2 and Cd73 at the plasma membrane (Fig. 3). As expected, RGF and RGF-N2 cells also express SV40 large antigen, which exhibits an expected exclusive nuclear localization (Fig. 3). Taken together, these results strongly indicate that both RGF and RGF-N2 cell lines express myofibroblast-specific markers and, do not derive from hepatocytes, hepatic stellate cells or cholangiocytes. Third, RGF and RGF-N2 cells were tested for DNA transfectability with an expression vector for monomeric green fluorescent protein (mGFP) with commercially available Fugene6 and Lipofectamine2000 reagents, according to manufacturer’s instructions. Immunofluorescence microscopy experiments indicate that DNA transfer is achievable in both RGF and RGF-N2 cells using Fugene6 reagent (DNA/reagent 1:3 ratio tested), as mGFP expression in transfected cells is clearly observed (Fig. 4). In contrast, transfection assays with lipid-based Lipofectamine2000 reagent were unsuccessful in the selected experimental conditions (DNA/reagent 1:6 ratio tested, not shown). These results demonstrate that DNA transfer can be successfully achieved in both RGF and RGF-N2 cell lines. Fourth, the capacity of RGF and RGF-N2 cells to inhibit bile ductular proliferation was examined, as a previous report by our group showed that loss of Ntpdase2 expression in primary isolated aPF culture allows increased bile duct cell proliferation in a co-culture assay[21]. Furthermore, regulation of bile duct cell proliferation could be restored, if Ntpdase2 expression was re-established in activated PF[21]. Because, RGF and RGF-N2 cells have been immortalized in an “early activation” step, at which Ntpdase2 expression was still observable, it is plausible that both cell lines still possess the ability to block bile ductular proliferation. Therefore, this hypothesis was tested, using an experimental setup similar to the one previously described above. Indeed, co-culture assays demonstrate that both RGF and RGF-N2 cell lines can significantly reduce the proliferation rate of bromodeoxyuridine-labeled Mz-Cha-1 cholangiocytes *in vitro* with the same relative efficacy (Fig. 5), comparable to cultured primary isolated aPF (not shown).

## Discussion

We report, in the present paper, the generation and functional characterization of two novel rat SV40-immortalized PF cell lines: RGF and RGF-N2. The RGF cell line was established by immortalization of cultured primary isolated aPF, while the RGF-N2 cell line was established by immortalization of cultured Ntpdase2-sorted primary isolated aPF. We performed preliminary studies to screen RGF and RGF-N2 cell lines for expression of common liver myofibroblast-associated genes. RGF and RGF-N2 express classical pan-myofibroblast *α*SMA (mRNA and protein), Pdgf receptor-β(mRNA and protein), type I Collagen *α*1 (mRNA), and PF-specific Elastin (mRNA and protein), type XV Collagen *α*1 (mRNA) and Fibulin-2 (mRNA), an expression pattern also observed in cultured primary isolated aPF (mRNA). Importantly, RGF and RGF-N2 cell lack expression of HSC-specific Desmin and Lrat marker mRNAs. Both cell lines do express cholangiocyte Keratin 19 mRNA, although no corresponding protein expression was noted by immunoblot analysis, using a polyclonal pan cytoKeratin antibody (Fig. 2) and a monoclonal Keratin 7 (Keratin 19 partner) antibody (not shown). We also observed differences in: 1) Vegfr2 mRNA expression between RGF (detected) and RGF-N2 (absent or weak) cell lines; 2) of Gpbar1 mRNA expression between cultured primary isolated aPF (detected) and RGF and RGF-N2 cell lines (weak); and 3) Gfap mRNA expression between cultured primary isolated aPF (absent) and RGF and RGF-N2 cell lines (detected). In both cases of *Vegfr2* and *Gbpar1* genes, we did not investigate the corresponding protein expression, for lack of reliable immunoblot-capable antibodies. In the case of *Gfap* gene, Gfap protein was expressed in early passages after immortalization but gradually decreased (after 45 passages). We sought to test the achievability of DNA transfer in RGF and RGF-N2 cell lines, which is an important feature to perform functional studies. Using a commercially-available transfection reagent, both RGF and RGF-N2 were found to be transfectable with DNA (and likely RNA, even though it was not tested here). Lastly, we demonstrated Ntpdase2-expressing RGF and RGF-N2 cell lines have the ability to block proliferation of Mz-ChA-1 cholangiocarcinoma cells, as was previously demonstrated for rat cultured primary isolated aPF. Importantly, we obviously cannot claim that the described polyclonal cell lines are functionally representative of all types of activated liver myofibroblasts originating from portal areas of the fibrosing liver. Specificity and reliability of cell markers, currently used to define cellular sources of liver myofibroblasts including portal fibroblasts, are still debated[1,4,33,34]. It is well possible that discrepancies in cell marker expression between species[35–38], as well as activation stage[8] may exist. Thus, these are critical parameters that need careful consideration if comparisons are to be made between RGF and RGF-N2 cell lines and portal liver myofibroblasts from other origins (likely mouse and human species). In summary, we have generated two novel cellular models for the *in vitro* study of liver portal (myo)fibroblasts functions.

## Supporting Information

S1 Fig Cultured primary isolated portal fibroblasts, and both immortalized RGF and RGF-N2 cells by phase contrast illumination microscopy. (TIF)

S2 Fig Characterization of primary isolated portal fibroblasts by cell sorting. (TIF)
