# Supplementary figures and images for: Establishment and Characterization of Rat Portal Myofibroblast Cell Lines
Source: PLoS One. 2015 Mar 30;10(3):e0121161. doi: 10.1371/journal.pone.0121161 (PMC4378927; doi:10.1371/journal.pone.0121161)

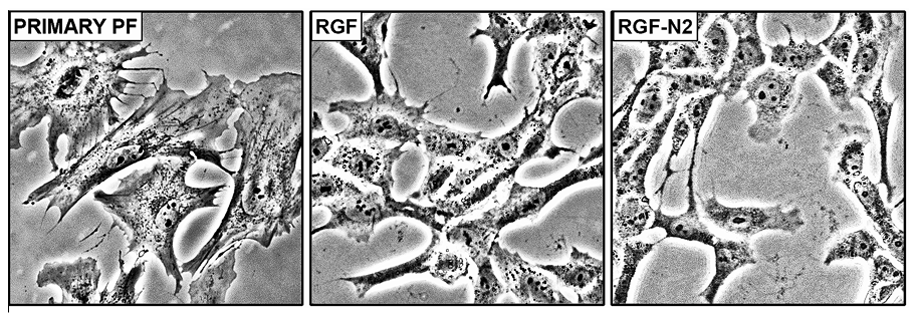

Supplement: S1 Fig — (TIF) [file pone.0121161.s001.tif]

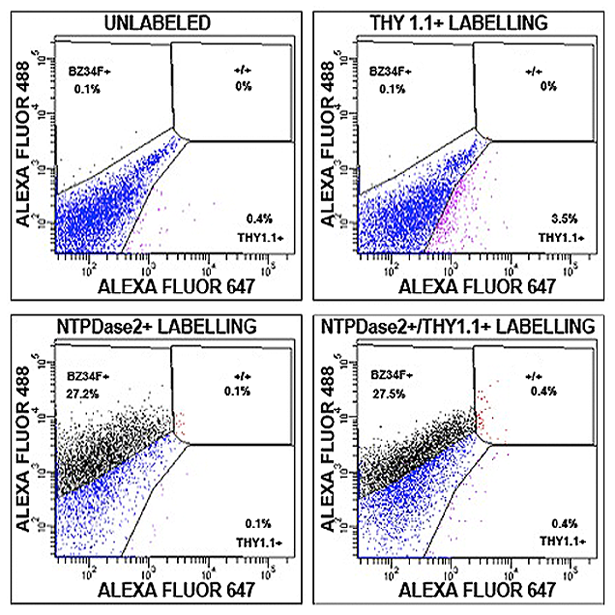

Supplement: S2 Fig — (TIF) [file pone.0121161.s002.tif]
